# A New Medical Dictionary

**Published:** 1890-08

**Authors:** 


A NEW MEDICAL DICTIONARY. Including all the Words and Phrases used in Medicine, with the proper Pronunciation and Definitions. Based on Recent Medical Literature. By GEORGE A. GOULD, B A , M. D., Ophthalmic Surgeon to the Philadelphia Hospital, and Clinical Chief Ophthalmological Department, German Hospital, Philadelphia. With Elaborate Tables of the Bacilli, Micrococci, Leucomaines, Ptomaines, etc.; of the Arteries, Ganglia, Muscles, Nerves and Plexuses, of Weights and Measures, Thermometers, etc; and appendices containing Classified Tables with Analysis of the Waters of the Mineral Springs of the United States, and Tables of Vital Statistics. Cloth, 8vo, pp. xi.—519. Philadelphia: P. Blakiston, Son & Co. 1890.

For a long time we were without good medical dictionaries, and the want was a serious one. Recently, however, owing to the labors of Foster and Billings, the want has been supplied in a most elaborate manner. These columns have contained notices of their works, and while our satisfaction with them, as already expressed, is very great, there seems still a place for a smaller and cheaper work (as far as price was concerned) that could be placed in the hands of students and others who might not be able to obtain the former. This place has been taken by the work of Dr. Gould, and there is no longer any excuse for a library to be without a good modern dictionary. He says truly in the preface that "it would not have been half the labor to make a volume double or treble its size;" the difficulty was to make a book compact, concise while omitting nothing of positive value. This, we think, the author has accomplished, and we can therefore say that it is the best work of its size that we know. We can recommend it without hesitation. The press-work is good — type, pages, and its general arrangement being very satisfactory. F. H. P.

